# Retraction Note: Circular RNA profiling reveals an abundant circLMO7 that regulates myoblasts differentiation and survival by sponging miR-378a-3p

**DOI:** 10.1038/s41419-026-09250-7

**Published:** 2026-09-07

**Authors:** Xuefeng Wei, Hui Li, Jiameng Yang, Dan Hao, Dong Dong, Yongzhen Huang, Xianyong Lan, Martin Plath, Chuzhao Lei, Fengpeng Lin, Yueyu Bai, Hong Chen

**Affiliations:** 1 Shaanxi Key Laboratory of Molecular Biology for Agriculture, College of Animal Science and Technology, Northwest A&F University, Yangling, 712100 Shaanxi China; 2 Bureau of Animal Husbandry of Biyang County, Biyang, 463700 Henan China; 3 Animal Health Supervision of Henan Province, Bureau of Animal Husbandry of Henan province, Zhengzhou, Henan 450008 China

Retraction Note to: *Cell Death & Disease* https://doi.org/10.1038/cddis.2017.541, published online 26 October 2017

The Editors-in-Chief have retracted this article. After publication, it was brought to the attention of the publisher that three panels in Fig. 10a of this article appear to overlap with figure 4 of a paper by the same author group [1] and one panel is also published in another article by the same author group [2]. All three papers were under consideration at the same time and describe different experimental conditions. The authors were unable to provide a satisfactory response to the concerns raised, or all of their original raw data or ethics approval documents upon request. The Editor has lost confidence in the data and conclusions of this article.

Author Xuefeng Wei disagrees with this retraction. Authors Hui Li, Jiameng Yang, Dan Hao, Dong Dong, Yongzhen Huang, Xianyong Lan, Martin Plath, Chuzhao Lei, Fengpeng Lin, Yueyu Bai, and Hong Chen did not respond to correspondence from the Publisher about this retraction.
